# Streptococcus lutetiensis prosthetic shoulder infection assisting in the diagnosis of invasive adenocarcinoma of the colon

**DOI:** 10.1016/j.xrrt.2024.05.001

**Published:** 2024-05-10

**Authors:** Majed Alzahabi, Jamil Haddad, Shariff K. Bishai

**Affiliations:** a Department of Orthopedic Surgery, Mclaren Macomb, Mount Clemens, MI, USA; b Department of Orthopedic Surgery, Henry Ford Macomb, Shelby Township, MI, USA; c Detroit Orthopaedic Institute, Troy, MI, USA

**Keywords:** Prosthetic joint infection, Streptococcus bovis group, Colorectal carcinoma, Streptococcus lutetiensis, Reverse total shoulder arthroplasty, Revision arthroplasty

The human gastrointestinal tract contains between 10^12^ and 10^14^ micro-organisms, predominantly bacteria, along with viruses, parasites, and fungi.^22^^,^^28^ Changes in the gut microbiota's composition occurs with modifications in diet, age and lifestyle in addition to variances in race and sex.^28^ There have been established disruptions and alterations in the microbiome community pattern of the gut in patients with colorectal cancer (CRC).^8^^,^^29^ Streptococcus bovis may possibly be equivalent to Streptococcus equinus, and have become grouped together as the Streptococcus bovis/Streptococcus equinus complex.^5^ Further division of the Streptococcus bovis group (SBG) is in groups of biotypes I and II.^7^^,^^17^ Sub-biotype II/1 is nominally referred to as Streptococcus lutetiensis.^21^ Streptococcus lutetiensis has been established in the literature as a rare cause of bacteremia.^15^ It is known to be associated with colon carcinoma with greater incidences of this subtype in patients with CRC in comparison to controls.^1^,^14^^,^^17^ It can cause disruptions in the colonic mucosa which can cause seeding of the bacteria throughout the body.^10^^,^^29^ Blood cultures may be negative, but the patient may still have culture-negative sepsis or a seeding of the bacteria without symptoms of sepsis.^24^ Streptococcus bovis bacteremia has been established as an indicator of underlying CRC through the means of chronic inflammation and lesions in the bowel and contain certain virulence factors which propagate carcinoma, but the specific mechanism is not well understood.^6^ Additionally, Streptococcus bovis is a rare cause of orthopedic infections, which can be caused secondary to bacteremia originating from the gastrointestinal tract.^18^ There can also be subclinincal levels of bacteria in the blood that, when in contact with the prosthetic, forms a biofilm on the foreign material and colonizes, resulting in an infection even with negative blood cultures.^19^ The specific linkage of Streptococcus bovis biotype II/1 or Streptococcus lutetiensis with prosthetic infections is, however, limited in the literature.

Anatomic shoulder replacements have been a very successful treatment of shoulder glenohumeral arthritis with intact rotator cuff muscles. However there are multiple risks associated with any orthopedic surgical procedures, including infection. Infection rates following primary total shoulder arthroplasty (TSA) range from <0.1% to 3.9%.^20^ The most common organism is *Cutibacteriumacnes* is the predominant organism (60.0%), followed by coagulase negative Staphlococcus (10.8%), Staphlococcus aureus (6.9%), or Staphylococcus epidermidis (2.0%).^13^ However, 18.3% of cases include various other organisms.^13^ Oftentimes, these organisms are inoculated into the joint incidentally through local inoculation intraoperatively during the incision.^19^ In fact, preoperative skin cultures can often help predict the organism in the case of periprosthetic infections in revised shoulder arthroplasties.^19^ This can help the surgeon attain antibiotic sensitivities for the local organisms, so that appropriate narrow antibiotics can be used promptly without the use of broad-spectrum antibiotics.^19^ While the most predominant organism is Cutibaceterium in shoulder infections, atypical organisms should promote a high clinical suspicion of another pathology.^13^ Specifically, infection with one of the subtypes of group D Streptococcus species, Streptococcus lutetiensis, should prompt a high suspicion of colorectal malignancy.^22^ The literature shows Streptococcus lutetiensis to be a rare cause of prosthetic joint infections.^13^ The association is well recognized with the presence of Streptococcus bovis bacteremia and unrecognized colonic malignancy.^26^ Awareness of this association helped prompt treatment of the CRC and 2-stage revision arthroplasty of the shoulder replacement.

In this report, we present the importance of screening of CRC in patients found to have Streptococcus lutetiensis periprosthetic joint infection (PJI). To our knowledge, there are currently no existing reports establishing the linkage of an orthopedic PJI of Streptococcus lutetiensis and the discovery of asymptomatic CRC.

## Case report/patient presentation

The patient was a 72-year-old man who presented in November of 2022 to the emergency department for evaluation of acute severe right shoulder pain, worsening over the past few days. The patient denied any traumatic injuries to the region. The patient had no cancer history, no abdominal complaints, no recent weight loss, no gastrointestinal bleeding and no changes to bowel habits. They had a negative family history of any gastrointestinal (GI) cancer. They did have a medical history of alcohol abuse, chronic obstructive pulmonary disease, coronary artery disease, abdominal aortic aneurysm. Previous routine colonoscopy within the past decade was negative for any abnormalities. They had a surgical history of an arthroscopic right shoulder cuff repair in 2013 followed by an anatomic TSA in October of 2016.

Physical examination revealed active forward flexion of 90°, active external rotation 10° past neutral and active internal rotation to the iliac crest.

**Figure 1:**
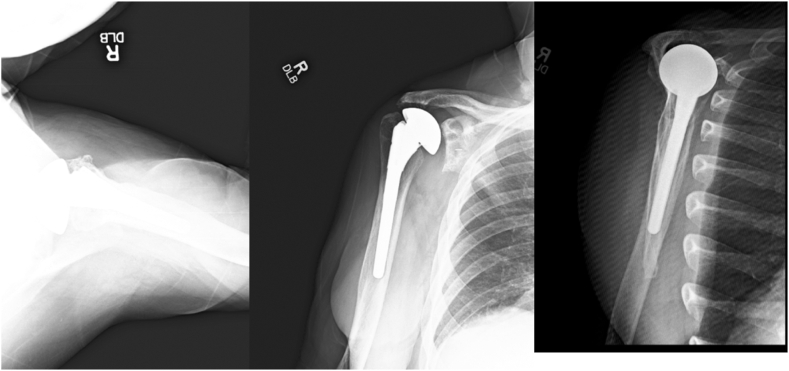
Radiographs revealing the original anatomic shoulder arthroplasty with in place TSA with lucencies around the glenoid component, indicating glenoid loosening. There is also superior migration of the humerus. *TSA*, total shoulder arthroplasty.

X-ray revealed the TSA in place with radiolucencies around the glenoid component, indicating glenoid loosening, in addition to rotator cuff failure shown from a superior migration of the humerus (Fig. 1).

Due to elevated inflammatory markers and shoulder pain, aspiration of the shoulder was done, which demonstrated calcium pyrophosphate disease. Cultures of the fluid were sent, but no antibiotics were started at discharge at that time. Cultures eventually grew Streptococcus lutetiensis, which prompting a consult to the infectious disease (ID) department for the atypical organism and they recommended a workup for CRC. This was recommended by ID due to the SBG subtype, Streptococcus lutetiensis, and its clear association with underlying colorectal carcinoma in the literature. During this same time period, the patient was sent for a colonoscopy which had polyps suspicious for invasive adenocarcinoma.

**Figure 2:**
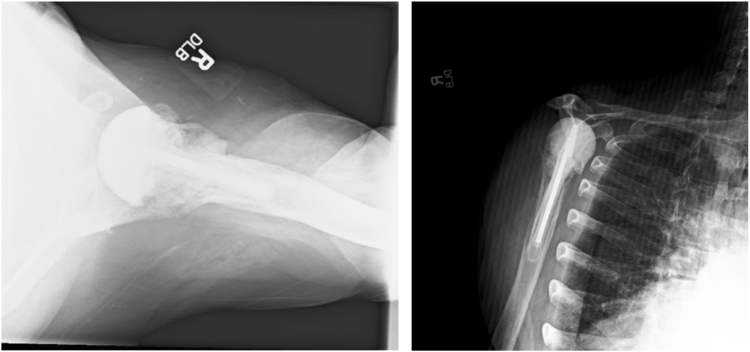
Radiographs revealing explanation of the anatomic shoulder arthroplasty and insertion of the antibiotic spacer.

The patient was then scheduled for a 2-stage revision with an explantation of right shoulder implants and antibiotic spacer placement on November 29, 2022. During the open irrigation, débridement, and explantation of implants, many tissue cultures were able to be obtained and they were negative. The subscapularis and supraspinatus tendons were noted to be torn. Serous fluid was noted with no frank pus. Care was taken to remove the humeral stem with minimal bone loss. The glenoid component was also removed with further soft tissue débridement. The antibiotic cement hemiarthroplasty was then placed with the shoulder noted to be stable throughout the range of motion testing (Fig. 2).

**Figure 3:**
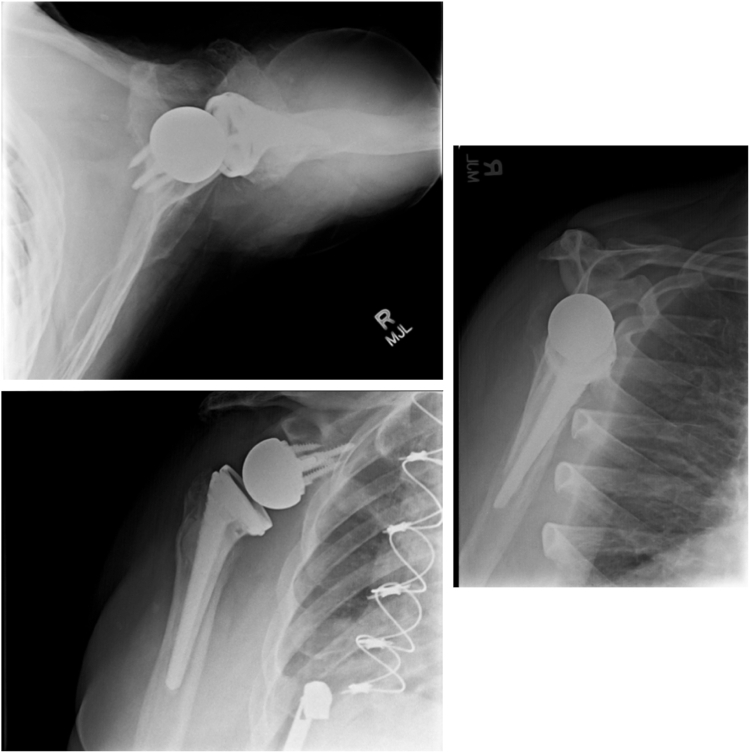
Radiographs of the final reverse total shoulder arthroplasty.

Intraoperative cultures demonstrated Streptococcus lutetiensis with good sensitivity to vancomycin. The patient was started on intravenous (IV) antibiotics for 6 weeks and then transitioned to oral clindamycin for 6 months with no antibiotic holiday. The patient was then recommended to have the final revision reverse TSA. ID did also recommend another 6 weeks of IV antibiotics after revision shoulder arthroplasty was placed to prevent any contamination or relapse of infection, as indolent organisms still needed to be eliminated (Fig. 3). The special consideration of the prolonged course of antibiotics was essential for the eradication of any of the Streptococcus lutetiensis microbes remaining in the gut.

As for the colon cancer, the patient was able to undergo right hemicolectomy which displayed negative margins for the adenocarcinoma, invading to the level of the submucosa. Further testing demonstrated no metastasis and patient colectomy helped prevent any further spread of the disease. Patient cleared the infection and is doing well today.

## Discussion

SBG were reported in intestinal flora among of 55% with colon carcinoma and 19% of those with noncolonic neoplasms.^25^ SBG can be part of the normal GI flora in 5%-16% of adults.^2^^,^^16^ However, colorectal carcinoma alters the native gut microbiomone, promoting Streptococcus bovis.^9^ The inflammatory proteins inhibit apoptosis, increase angiogenesis and cause local areas of chronic inflammation, contributing to neoplasm formation in certain cases.^3^^,^^12^ Streptococcus bovis PJI have been found, at some institutions, to be as low as 0.4% of all PJIs. Streptococcus lutetiensis is a rare entity in shoulder periprosthetic surgeries.^13^ One particular mechanism upon which Streptococcus lutetiensis can spread to a joint is a GI mucosal tract abnormality predisposing to bacterial translocation of the bacteria into the bloodstream, then infiltrating and forming a biofilm on a prosthetic joint, which is the method in which bacteremia of Streptococcus bovis occurs.^23^ The specific mechanism in which it is associated with colonic neoplasms and progresses to bacteremia and local areas of infection has been an area of ongoing study.^4^ Streptococcus bovis is more commonly implicated as a pathogen in infection in the colon. Any diagnosis of Streptococcus bovis endocarditis for the European Society of Cardiology entails a colonoscopy during hospitalization, and annual repeats, if negative.^11^ No such recommendation exists, to our knowledge, with Streptococcus lutetiensis PJI and colonoscopies.

CRC is currently the fourth most prevalent cancer in the United States with a 5-year survival rate of 65%.^23^ In spite of its relatively good prognosis, it can be further improved by the early treatment of polyps and colorectal malignancies have a positive impact on survival.^5^^,^^27^ All things considered, a SBG PJI may assist in the early diagnosis of CRC, with a quicker referral to treatment and less time for metastatic spread and poorer prognoses.

Prosthetic joint infections are a known and devastating complication of any joint replacement. Knowing the common organisms and having a high index of suspicion of further pathology is essential for helping guide proper treatment of your patients. This patient had an uncomplicated anatomic shoulder arthroplasty over 6 years before his new onset shoulder pain. He also had no known signs or symptoms of colon adenocarcinoma. His prompt diagnosis helped expedite treatment and surgical excision of the local, malignant carcinoma that had not yet spread. This prevented the progression of the asymptomatic colorectal adenocarcinoma from metastasizing or growing further until abdominal symptoms became noticed or a routine colonoscopy performed.

Streptococcus lutetiensis is 1 of the 4 subtypes of Streptococcus bovis. Streptococcus bovis is a known bacteria in healthy individuals but in bacteremia or infection has had known associations with CRC. However, any subtype in the class should prompt further workup with colonoscopy and proper imaging.

## Conclusion

Shoulder periprosthetic infections are a feared complication following operative replacement of the native shoulder. Infection of the shoulder should warrant high suspicion of a potential bacteremic spread, if cultures result in atypical organisms, not associated with normal skin flora or common contaminants. Specifically, in the consideration of an organism in the SBG, such as Streptococcus lutetiensis, appropriate referral to gastroenterologists for the workup of potentially indolent colorectal malignancy should be made. Prompt referral after culture results with earlier diagnosis of a potential underlying malignancy can result in a better outcome.

## Disclaimers:

Funding: No funding was disclosed by the authors.

Conflicts of interest: The authors, their immediate families, and any research foundations with which they are affiliated have not received any financial payments or other benefits from any commercial entity related to the subject of this article.

Patient Consent: Obtained.
